# Directed differentiation of human induced pluripotent stem cells into mature stratified bladder urothelium

**DOI:** 10.1038/s41598-019-46848-8

**Published:** 2019-07-19

**Authors:** Kotaro Suzuki, Michiyo Koyanagi-Aoi, Keiichiro Uehara, Nobuyuki Hinata, Masato Fujisawa, Takashi Aoi

**Affiliations:** 1 Division of Advanced Medical Science, Graduate School of Science, Technology and Innovation, Kobe University, Kobe, Japan; 2 Department of iPS cell applications, Graduate School of Medicine, Kobe University, Kobe, Japan; 3 Division of Urology, Graduate School of Medicine, Kobe University, Kobe, Japan; 4 Center for Human Resource development for Regenerative Medicine, Kobe University Hospital, Kobe, Japan; 5 Division of Pathology, Graduate School of Medicine, Kobe University, Kobe, Japan

**Keywords:** Pluripotent stem cells, Stem-cell differentiation

## Abstract

For augmentation or reconstruction of urinary bladder after cystectomy, bladder urothelium derived from human induced pluripotent stem cells (hiPSCs) has recently received focus. However, previous studies have only shown the emergence of cells expressing some urothelial markers among derivatives of hiPSCs, and no report has demonstrated the stratified structure, which is a particularly important attribute of the barrier function of mature bladder urothelium. In present study, we developed a method for the directed differentiation of hiPSCs into mature stratified bladder urothelium. The caudal hindgut, from which the bladder urothelium develops, was predominantly induced via the high-dose administration of CHIR99021 during definitive endoderm induction, and this treatment subsequently increased the expressions of uroplakins. Terminal differentiation, characterized by the increased expression of uroplakins, CK13, and CK20, was induced with the combination of Troglitazone + PD153035. FGF10 enhanced the expression of uroplakins and the stratification of the epithelium, and the transwell culture system further enhanced such stratification. Furthermore, the barrier function of our urothelium was demonstrated by a permeability assay using FITC-dextran. According to an immunohistological analysis, the stratified uroplakin II-positive epithelium was observed in the transwells. This method might be useful in the field of regenerative medicine of the bladder.

## Introduction

Patients with bladder congenital anomalies, neuropathic disorder, chronic inflammation, and bladder cancer undergo cystectomies and subsequently often require augmentation or reconstruction of the urinary bladder. Gastrointestinal tissue has generally been employed as a substitute for bladder tissue in these cases. While this approach does provide a urinary reservoir function, the use of intestinal tissue for this purpose often leads to significant complications, including recurrent infections, bladder stones, metabolic disturbance, and an increased cancer risk^1,2^. Furthermore, patients are at risk for perioperative complications due to this approach, including bowel obstruction and bowel leak of anastomosis^3^.

To overcome these complications, autologous bladder cells have been studied as alternative biomaterials for augmentation or reconstruction of the urinary bladder. Many reports in animal models have examined the utility of autologous bladder cells for bladder augmentation^4^. Furthermore, in a human clinical study for pediatric patients with neurogenic bladders treated with autologous bladder cells seeded onto collagen-coated polyglycolides, most of the patients showed improved bladder compliance and capacity without the complications associated with the intestinal tissue approach after up to 61 months of follow-up^5^. However, it is generally difficult to maintain and expand autologous cells^6^. In addition, bladder cells from diseased bladder are not ideal because of the risk of cancer recurrence or an impaired proliferative ability of the cells^7–9^.

Thus, bladder urothelium derived from adult stem cells or pluripotent stem cells, including human embryonic stem cells (hESCs) and human induced pluripotent stem cells (hiPSCs), have received focus as alternative sources^4,7,10^. hiPSCs are particularly attractive candidates, as they can overcome problems associated with the autologous cells approach thanks to their ability to be induced from any cell type and proliferate infinitely^11^. Many reports have found that hiPSCs can differentiate into various somatic cells. In some of these reports, the recapitulation of the developmental process using a combination of several small molecules improved the efficiency of differentiation and induced more mature cells from hiPSCs^12–14^.

Although several reports on the differentiation of hiPSCs into bladder urothelium have reported that these cells can differentiate into uroplakin-positive cells^15–17^, the efficiency of the differentiation and the maturation of these cells has yet to be evaluated. The stratified structure is a particularly important attribute of the mature bladder urothelium because the structure plays a critical role in the bladder providing a barrier against pathogens, toxins and waste products in urine^18,19^; however, no report has demonstrated the derivation of stratified bladder urothelium from hiPSCs.

In the present study, we developed a novel protocol for the directed differentiation of hiPSCs into stratified bladder urothelium through the posterior definitive endoderm (DE) and caudal hindgut by recapitulating embryogenesis using high-dose CHIR99021. In addition, we demonstrated the enhanced terminal differentiation and stratification of bladder urothelium derived from hiPSCs using a combination of a PPAR-γ agonist and an EGFR inhibitor as well as FGF10 and transwell culture.

## Results

### High-dose GSK3β inhibitor treatment led first to posterior DE and then caudal hindgut differentiation

Bladder urothelial cells are derived through posterior DE, caudal hindgut, urogenital sinus^20,21^, and terminal differentiation in embryogenesis. Although several previous reports have suggested that a high dose of GSK3β inhibitor enhanced the directed differentiation of several hiPSC lines to the posterior DE^22–24^, the concrete values of the concentrations and durations of treatment varied among the reports, suggesting that the optimal concentration and duration of GSK3β inhibitor treatment differ among cell lines. We therefore attempted to optimize the GSK3β inhibitor treatment conditions for the hiPSC line confirmed to be pluripotent (Fig. S1A–D) and which was used in this study.

First, to clarify the maximum tolerable dose of the GSK3β inhibitor CHIR99021, we added 1, 3, 5, or 7 μM of CHIR99021 to the medium for 3 days of DE induction with Activin A (Fig. S2A). CHIR99021 was obviously toxic at 7 µM (Fig. S2B) and resulted in the reduced expression of the endoderm marker SOX17 (Fig. S2C). At concentrations between 1 and 5 μM, we found that the expression of CDX2, a marker of posterior DE, was increased in a dose-dependent manner with no obvious difference in the SOX17 expression (Fig. S2C). We therefore adopted 5 μM as the high dose and 1 μM as the low dose of CHIR99021 treatment in the present study.

**Figure 1:**
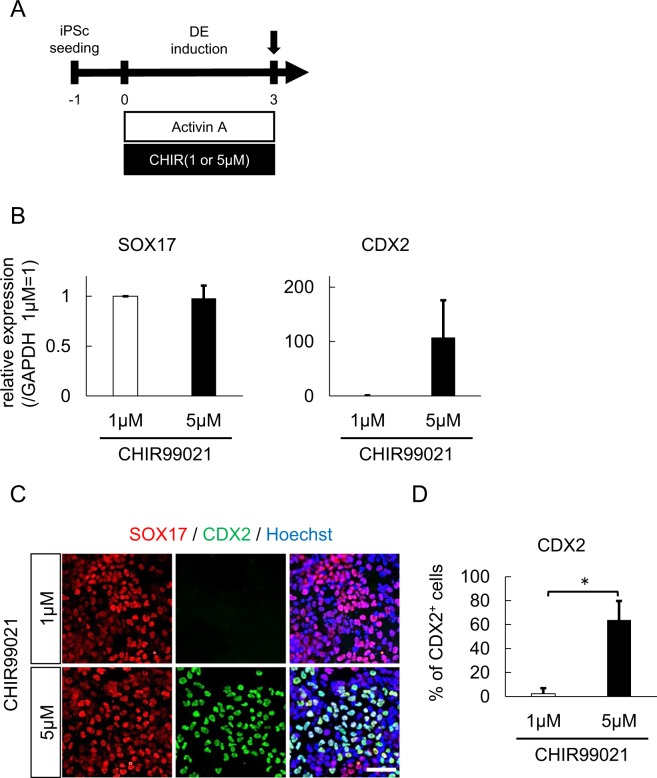
Treatment with a high-dose GSK3β inhibitor predominantly induced posterior DE. (**A**) A schematic diagram of posterior DE induction using Activin A and CHIR99021. (**B**) An expression analysis of SOX17 and CDX2 in differentiated cells treated with the indicated dose of CHIR by qPCR (n = 3 independent experiments; mean ± SEM). (**C**) Immunostaining of SOX17 and CDX2 in differentiated cells. Representative fluorescence microscopic images for treatment with 1 µM (upper panels) and 5 µM CHIR (lower panels) are shown. Scale bar, 50 μm. (**D**) The proportion of CDX2-positive cells after treatment with 1 or 5 µM CHIR (n = 3 independent experiments; mean ± SD). *p < 0.05; two-tailed paired *t*-test. Abbreviations: iPSc, induced pluripotent stem cell; DE, definitive endoderm; CHIR, CHIR99021.

To confirm the efficacy of high-dose CHIR99021 in posterior DE induction from hiPSCs, we quantitatively compared the differentiation efficiency between 1 μM and 5 μM CHIR99021 in 3 independent experiments (Fig. 1A). The expression of CDX2 mRNA was about 100-fold higher in 5 μM than 1 μM, while the expression of SOX17 mRNA was similar in both conditions (Fig. 1B). A comparative immunocytochemistry analysis showed that the expression of CDX2 protein was enhanced by 5 μM of CHIR99021 treatment, while the expression of SOX17 was roughly unaffected (Fig. 1C). The mean proportions of CDX2 + cells were 2.3% and 63.8% with 1 μM and 5 μM of CHIR99021 treatment, respectively (Fig. 1D). In contrast to a previous report showing 1-day treatment of CHIR99021 to be effective^22^, 1-day treatment of CHIR99021 did not enhance the CDX2 expression in the present study (Fig. S2D,E). On the other hand, the expression of SOX2 mRNA was increased in the cells treated with 1 µM CHIR99021 (Fig. S2F), suggesting that these cells were predominantly differentiated into anterior DE.

**Figure 2:**
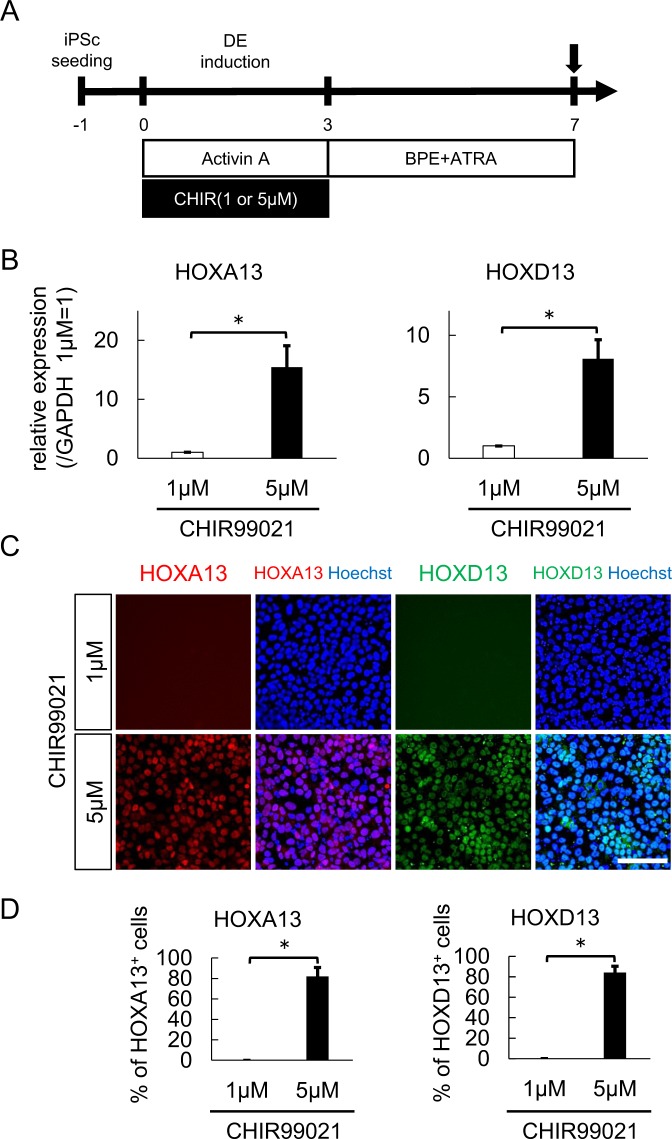
Treatment with a high-dose GSK3β inhibitor efficiently induced caudal hindgut. (**A**) A schematic diagram of hindgut maturation after posterior DE induction. (**B**) An expression analysis of the hindgut markers HOXA13 and HOXD13 after four days of maturation following posterior DE induction with the indicated dose CHIR by qPCR (n = 3 independent experiments; mean ± SEM). (**C**) Immunostaining of HOXA13 and HOXD13 in differentiated cells. Representative fluorescence microscopic images for treatment with 1 µM (upper panels) and 5 µM CHIR (lower panels) are shown. Scale bar, 100 µm. (**D**) The proportions of HOXA13 (left panel)- and HOXD13 (right panel)-positive cells (n = 3 independent experiments; mean ± SD). *p < 0.05; two-tailed paired *t*-test. Abbreviations: iPSc, induced pluripotent stem cell; DE, definitive endoderm; CHIR, CHIR99021; BPE, bovine pituitary extract; ATRA, all-trans retinoic acid.

To induce the differentiation of the posterior DE cells toward bladder urothelium, we employed keratinocyte serum-free medium containing bovine pituitary extract (BPE) and all-trans retinoic acid (ATRA), in reference to previous reports^15,16^. We evaluated the effect of CHIR99021 treatment on the expression of HOXA13 and HOXD13, which are essential inductive signals for the normal development of the hindgut^25–28^, four days after posterior DE induction had ended (Fig. 2A). In the quantitative polymerase chain reaction (qPCR) analysis, 5 μM CHIR99021 treatment increased the expression of HOXA13 (15.4-fold, p < 0.05) and HOXD13 (8.1-fold, p < 0.05) compared to 1 μM treatment (Fig. 2B). In a comparative immunocytochemistry analysis of HOXA13+ and HOXD13+ cells, while there were no positive cells after 1 μM treatment, 5 μM treatment induced positivity in 81.9% of HOXA13 and 84.2% of HOXD13 cells (Fig. 2C,D).

### A PPAR-γ agonist and an EGFR inhibitor enhanced the terminal urothelial differentiation from hiPSCs

**Figure 3:**
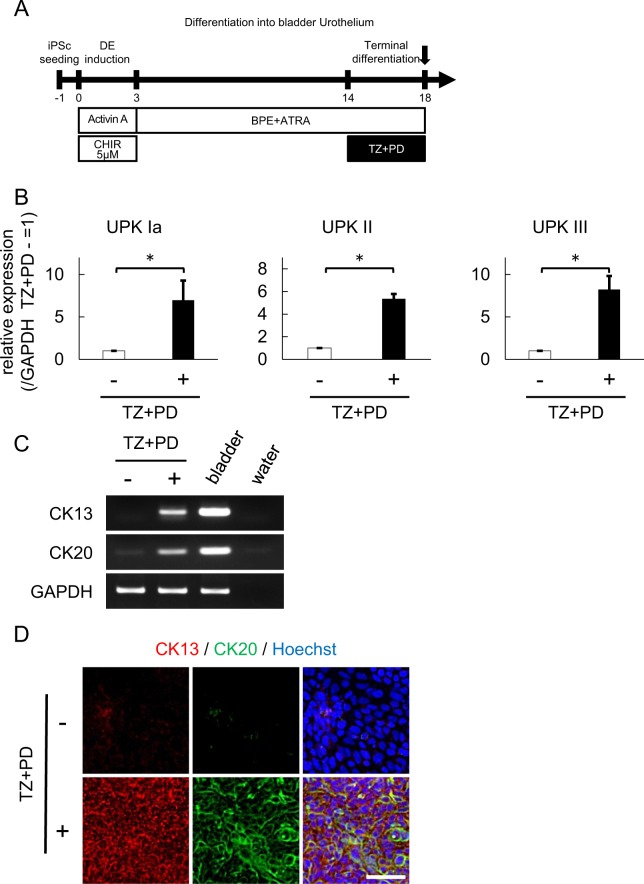
A PPAR-γ agonist and an EGFR inhibitor enhanced the terminal transitional differentiation into bladder urothelium. (**A**) A schematic diagram shows that the PPAR-γ agonist Troglitazone (TZ) and EGFR inhibitor PD153035 (PD) were added for the last four days to complete terminal transitional differentiation into bladder urothelium. (**B**) A qPCR analysis of the urothelial markers UPK Ia, UPK II, and UPK III after TZ + PD treatment for 4 days to complete terminal differentiation (n = 5 independent experiments; mean ± SEM, *p < 0.05; two-tailed paired t test). (**C**) An expression analysis of the transitional differentiation markers CK13 and CK20 at day 18 by semi-quantitative RT-PCR. Bladder RNA was used as a positive control. Full-length gels are presented in Supplementary Figure 6. (**D**) Immunostaining of CK13 and CK20 in differentiated cells after treatment with (lower panels) or without (upper panels) TZ + PD for 4 days. Representative fluorescence microscopic images are shown. Scale bar, 50 μm. Abbreviations: iPSc, induced pluripotent stem cell; DE, definitive endoderm; CHIR, CHIR99021; BPE, bovine pituitary extract; ATRA, all-trans retinoic acid; TZ, troglitazone; PD, PD153035; UPK, uroplakin.

Previous reports using primary urothelium culture have suggested that the combination of the PPAR-γ agonist troglitazone (TZ), and the EGFR inhibitor PD153035 (PD) (hereafter referred to as TZ + PD) enhances the terminal differentiation of bladder urothelium^29–33^. We thus evaluated whether or not the combination of TZ + PD enhances terminal urothelial differentiation from hiPSCs. We added 1 μM of TZ + PD during the last 4 days of differentiation for 18 days (Fig. 3A). qPCR revealed that the combination of TZ + PD increase the expression of the following urothelial markers: Uroplakin IA (7.0-fold, p < 0.05), II (5.6-fold, p < 0.05), and III (8.2-fold, p < 0.05) (Fig. 3B). Furthermore, semi-quantitative reverse transcription PCR (RT-PCR) (Fig. 3C) and immunocytochemistry (Fig. 3D) showed the mRNA and protein expressions of the terminal differentiation markers CK13 and CK20 induced by TZ + PD. In addition, we evaluated the effect of a single administration of TZ and PD. A semi-quantitative RT-PCR showed that the single administration of TZ also induced terminal differentiation. but the expression of CK13 and CK20 of these cells was markedly lower than in those treated with TZ + PD (Fig. S3).

**Figure 4:**
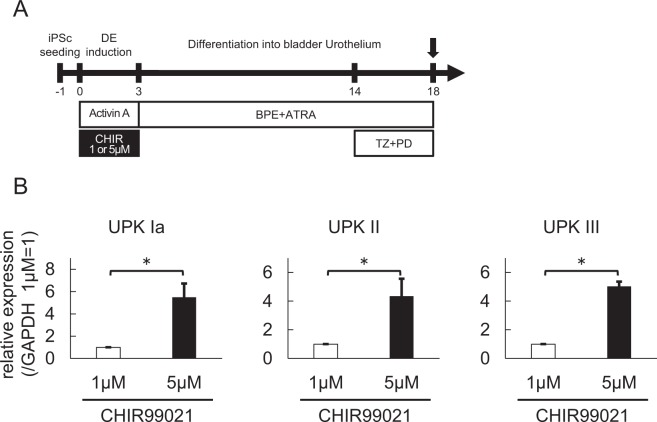
Treatment with a high-dose GSK3β inhibitor during posterior DE induction enhanced urothelial differentiation. (**A**) An overview of the culture protocol for urothelial differentiation. (**B**) An expression analysis of the urothelial markers in differentiated cells treated with a high (5 µM) or low (1 µM) dose of CHIR during posterior DE induction by qPCR (n = 5 independent experiments; mean ± SEM). *p < 0.05; two-tailed paired *t*-test. Abbreviations: iPSc, induced pluripotent stem cell; DE, definitive endoderm; CHIR, CHIR99021; BPE, bovine pituitary extract; ATRA, all-trans retinoic acid; TZ, troglitazone; PD, PD153035; UPK, uroplakin.

We further compared the expression of Uroplakin IA, II, and III after 18 days of differentiation using TZ + PD with 1 and 5 μM of CHIR99021 treatment during DE induction (Fig. 4A). The increased expressions of Uroplakin IA (5.0-fold, p < 0.05), II (4.3-fold, p < 0.05), and III (5.6-fold, p < 0.05) in the samples treated with 5 μM compared to those treated with 1 μM (Fig. 4B) indicated that a high-dose GSK3β inhibitor and TZ + PD had an additive effect on bladder urothelial differentiation from hiPSCs.

### FGF10 treatment enhanced the differentiation into stratified urothelium

FGF10 secreted from mesenchyme has been reported to play an important role in regulating the growth, differentiation, and repair of the urothelium in a paracrine manner^34,35^, and previous reports have shown that urothelium failed to stratify in Fgf10-null mice^36^.

**Figure 5:**
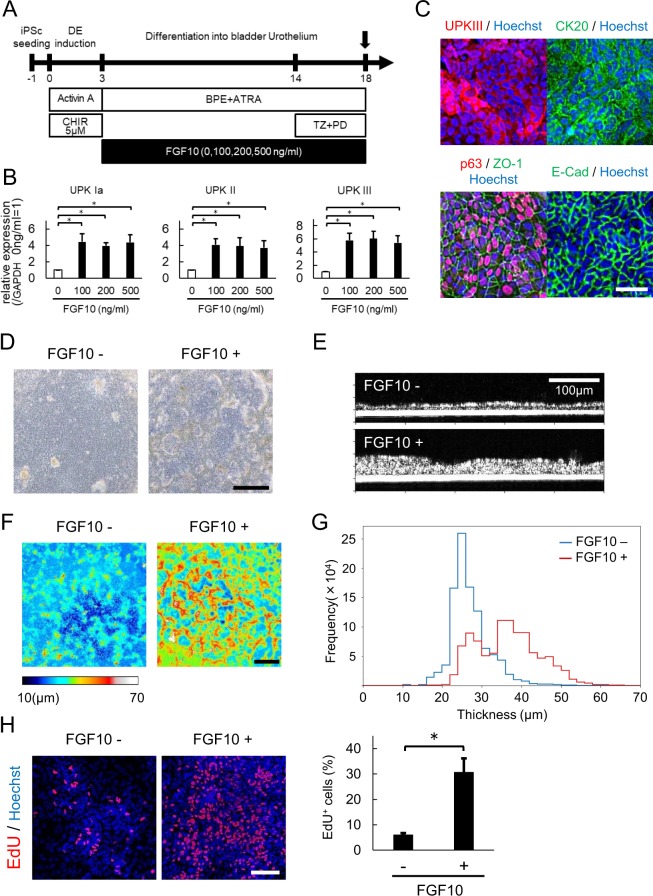
FGF10 enhanced urothelial differentiation and stratification. (**A**) After posterior DE induction, cells were treated with 0, 100, 200, or 500 ng/ml FGF10 from days 3 to 18. (**B**) A quantitative RT-PCR analysis of urothelial markers (UPK Ia, UPK II, and UPK III) in differentiated cells at day 18 after treatment with the indicated concentrations of FGF10. Data are shown as the mean ± SEM (n = 6 independent experiments). *p < 0.05; Dunnett’s test. (**C**) Immunostaining of UPK III, CK20, p63, ZO-1 and E-Cadherin in differentiated cells. Scale bar, 50 µm. (**D**) Phase contrast images of differentiated cells with (right panel) or without (left panel) FGF10 treatment (100 ng/ml). (**E**) Optical coherence tomography (OCT) images of differentiated cells treated with (lower panel) or without (upper panel) FGF10 in high-resolution mode. (**F**) The thicknesses of the differentiated cells with (right panel) or without (left panel) FGF10 treatment were visualized using a color-scale heatmap from 10 to 70 μm in low-resolution mode. Scale bar, 1 mm. (**G**) A histogram of the OCT analysis of differentiated cells with (red line) or without (blue line) FGF10 treatment in low-resolution mode. (**H**) An EdU assay for the cells with and without FGF10 treatment. Representative fluorescence microscopic images of EdU (left panel) and the proportions of EdU-positive cells (right panel) are shown (n = 5 different fields; mean ± SD). *p < 0.05; two-tailed paired *t*-test. Abbreviations: iPSc, induced pluripotent stem cell; DE, definitive endoderm; CHIR, CHIR99021; BPE, bovine pituitary extract; ATRA, all-trans retinoic acid; TZ, troglitazone; PD, PD153035; UPK, uroplakin.

We therefore examined whether or not FGF10 treatment enhances bladder urothelial differentiation from hiPSCs by adding various concentrations of FGF10 to the culture media for 2 weeks subsequent to DE induction (Fig. 5A). In a qPCR analysis, FGF10 treatment at concentrations of ≥100 ng/ml resulted in about a 5-fold increased expression of uroplakin IA, II, and III (Fig. 5B), indicating enhanced bladder urothelial differentiation. An immunocytochemistry analysis revealed the UPKIII protein expression at the cell membrane and cytoplasm after treatment with 5 μM of CHIR99021, TZ + PD, and FGF10 (Fig. 5C). Our urothelium also showed other urothelial markers, such as p63, CK20, ZO-1 and E-Cadherin, on immunostaining (Fig. 5C), but the expression of these markers’ mRNA was not enhanced by FGF10 (Fig. S4A).

Notably, FGF10 treatment seemed to increase the elevated regions in phase-contrast microscopy (Fig. 5D). In order to more objectively evaluate the thickness of differentiated cell clumps, we employed optical coherence tomography (OCT). OCT showed that the epithelium treated with FGF10 was actually thicker (by about three-fold) than that without FGF10 treatment (Fig. 5E). In the analysis of the distribution, the epithelium treated with FGF10 was generally thicker than that without FGF10 treatment, as the proportions of the areas with a thickness of ≥30 µm in the culture with and without FGF10 treatment were 19.8% and 71.2%, respectively (Fig. 5F,G). These findings suggest that FGF10 enhanced the stratification of urothelial cells. To clarify whether the effect of FGF10 was due to the promotion of cell proliferation or to the suppression of cell death, we performed an EdU assay and Caspase assay. As shown in Fig. 5H, the uptake of EdU was markedly enhanced with FGF10 treatment. The uptake of EdU was detected in the elevated regions. In contrast, there was no marked difference in the expression of cleaved caspase-3 between cells with and without FGF10 treatment (Fig. S4B). These findings suggested that stratification by FGF10 was due to the promotion of cell proliferation.

### Transwell culture enhanced the generation of stratified urothelium

In previous reports, a transwell culture system was used for the differentiation of hiPSCs into polarized and functional tissues^37–42^.

**Figure 6:**
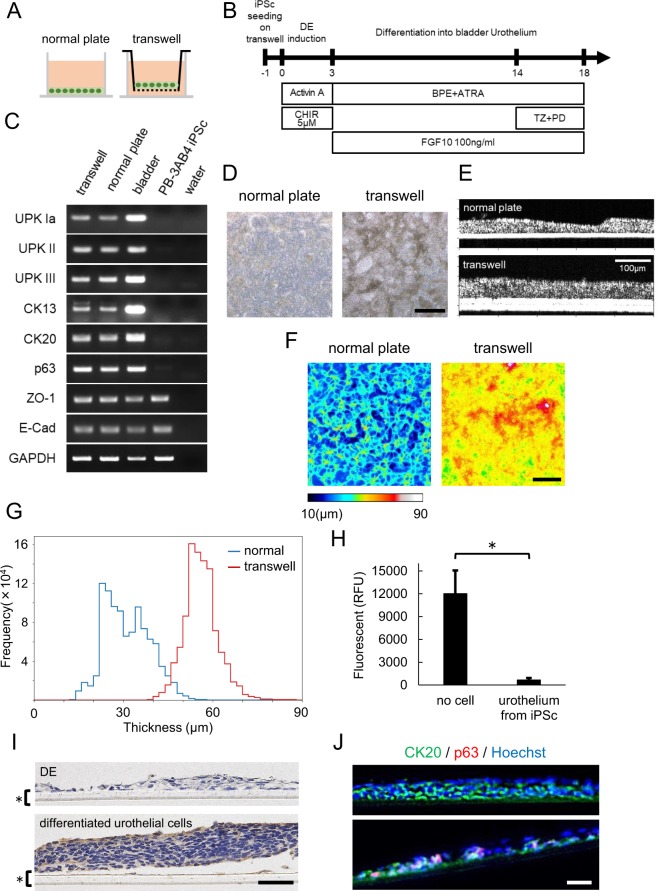
Transwell culture generated more stratified epithelium than normal plate culture. (**A**) Schematic images of normal plate culture (left) and transwell culture (right) for urothelial differentiation. (**B**) An overview of the culture protocol on transwell. (**C**) A semi-quantitative RT-PCR analysis of urothelial markers (UPK Ia, UPK II, UPK III), transitional differentiation markers (CK13, CK20) and other differentiation markers (p63, ZO-1 and E-Cadherin) in differentiated cells cultured in the normal plates and transwells. Total RNA of human bladder tissue was used as a positive control. Full-length gels are presented in Supplementary Figure 7. (**D**) Phase contrast images of differentiated cells at day 18 on normal plate (left panel) and transwell (right panel). Scale bar, 500 μm. (**E**) OCT images of differentiated cells on normal plate (upper panel) and transwell (lower panel). (**F**) The thicknesses of the differentiated cells on normal plate (right panel) or transwell (left panel) were visualized using a color-scale heatmap from 10 to 90 µm in low-resolution mode. Scale bar, 1 mm. (**G**) the histogram distribution of the OCT analysis of differentiated cells on normal plate (blue line) and transwell (red line) in low-resolution mode. (**H**) An *in vitro* permeability assay using FITC dextran. Fluorescence intensities were quantified by relative fluorescence units (RFU) (n = 3 independent experiments; mean ± SEM, *p < 0.05; two-tailed paired *t*-test). (**I**) Immunostaining of UPK II in DE cells (upper panel) and differentiated urothelial cells (lower panel) on a transwell plate (*). Scale bar, 50 μm. (**J**) Immunostaining of CK20 and p63 in differentiated urothelial cells in the stratified area (upper panel) and less-stratified area (lower panel). Abbreviations: iPSc, induced pluripotent stem cell; DE, definitive endoderm; CHIR, CHIR99021; BPE, bovine pituitary extract; ATRA, all-trans retinoic acid; TZ, troglitazone; PD, PD153035; UPK, uroplakin.

To examine the effect of transwell culture on our objective, we seeded hiPSCs onto a transwell as well as a normal plate (Fig. 6A) and carried out the urothelial differentiation protocol optimized in the present study (Fig. 6B). We used the same culture medium both above and below the transwell membrane. To clarify the presence of any difference at the DE and caudal hindgut stages between normal culture and transwell systems, we evaluated the expression of CDX2 at day 3 and the expression of HOXA13 and HOXD13 at day 7 in normal plates and transwell plates treated with 1 or 5 µM CHIR99021. On semi-quantitative RT-PCR, the cells showed similar levels of CDX2, HOXA13 and HOXD13 between the two systems (Fig. S5A,B), suggesting that there was no marked difference at the DE or caudal hindgut stage. We next confirmed the mRNA expression of uroplakins, CK13, CK20, p63, ZO-1 and E-Cadherin in the cells in the transwell and normal plates at day 18 (Fig. 6C). Notably, the epithelium in the transwell culture system elevated more extensively than that in normal plate culture in phase-contrast microscopic images (Fig. 6D). We again employed OCT, and it showed that the epithelium on transwell was actually thicker than that on normal plate (Fig. 6E). In the analysis of the distribution, the epithelium on the transwell was generally thicker than that on the normal plate, as the proportions of the areas with a thickness of ≥30 µm on the normal plate and transwell were 49.2% and 95.6%, respectively (Fig. 6F,G). These findings suggested that transwell culture further enhanced the stratification of urothelial cells differentiated with FGF10. Next, we performed an *in vitro* permeability assay using FITC dextran to evaluate the barrier function of our stratified urothelium from iPSC. As shown in Fig. 6H, our urothelium in transwell culture obviously blocked the permeation of FITC dextran, suggesting that our bladder urothelium from iPSCs has a viable barrier function. In an immunohistological analysis, stratified epithelial cells expressed uroplakin II (Fig. 6I) and CK20 (Fig. 6J) in all layers, despite these being markers of superficial urothelial cells. In contrast, cells expressing the basal cell marker p63 were detected in only less-stratified areas (Fig. 6J lower panel) and not in stratified areas at all (Fig. 6J upper panel). Although our urothelium did not identically reproduce an authentic bladder urothelial structure with the correct distribution of superficial and basal cells, these results indicated that our differentiation protocol with a transwell culture system provided stratified epithelial tissue structure which has barrier function resembling urothelium *in vivo*.

## Discussion

In the present study, we succeeded in the directed differentiation of hiPSCs into mature bladder urothelium through the posterior DE and caudal hindgut. While some previous reports have shown that a high dose of CHIR99021 enhanced the differentiation of hiPSCs into cells positive for some markers of the posterior DE as well as the mid hindgut and intestine^23,24^, which is one of the derivatives of posterior DE, this is the first report demonstrating the effect of CHIR99021 on the caudal hindgut and bladder urothelium. In addition to establishing a novel protocol to obtain human urothelium from iPSCs, our results provide evidence that the derivative of hiPSCs dominantly induced by high-dose CHIR99021 with Activin A actually recapitulate authentic posterior DE able to give rise to not only intestine but also lower urinary tract tissue. Furthermore, as a primary culture of urothelial cells, terminal differentiation of the present urothelium derived from hiPSCs was enhanced by TZ + PD^29–33^. This consistent responsiveness to TZ + PD suggested that our urothelium from iPSCs recapitulates authentic bladder urothelium; notably, no previous reports of iPSC-derived cells expressing urothelial markers have mentioned the responsiveness to TZ + PD. In the present study, we only demonstrated the positive effect of TZ + PD on urothelial differentiation, but did not specify the optimal addition timing and concentration of TZ + PD for the iPSC line used in the study; PB3AB4. Generally, the ideal administration timing and concentration of compounds for differentiation to a certain type of cells will differ among cell lines^43^. Therefore, the optimized conditions should be examined for each individual cell line by the researchers using the line.

It should be noted that we generated stratified structure of urothelium derived from hiPSCs through the use of FGF10 and a transwell culture system that permits the cells to take up the nutrient on basal surfaces, mimicking *in vivo* condition. One of the essential roles of the bladder is to function as a barrier against pathogens, toxins and waste products in urine^18,19^, and the stratification of bladder urothelium contributes to this barrier function. Thus, the stratification of bladder urothelium is very important for the future clinical application of this technique. In addition to the tissue functions, the ordered structures of tissues are generally implicated in the proliferation and differentiation of each individual component cell of the tissues in the development, regeneration and tumorigenesis in various kinds of tissues. Thus our stratified urothelium model might be applicable for research on the mechanisms underlying the development, regeneration, tumorigenesis, and tumor invasion of the human bladder.

The structure of the stratified urothelium derived from hiPSCs in the present study, in which all layers were positive for uroplakins, did not identically recapitulate an authentic bladder urothelial structure, in which the apical region is positive for uroplakins while the basal region is negative. This might be due to the incomplete recapitulation of FGF signaling in our present system. FGF10-FGFR2IIIb signal has been reported to play an important role in regulating the growth, differentiation, repair, and stratification of the urothelium^34,35^. FGF10 treatment was found to consistently enhance the expression of uroplakins, which are differentiation markers of bladder urothelium, and urothelial stratification in our study. Interestingly, FGF7, another ligand of FGFR2IIIb, also enhanced the stratification of urothelium in mice, similar to FGF10, but suppressed the differentiation of urothelium^44^, in contrast to FGF10. FGF7 is considered to originate in the mesenchyme and be active at the urothelium^45^. FGF7 from mesenchyme may thus cause the negative expression of uroplakins in the basal region of urothelium. Considering the distribution of superficial markers (uroplakins and CK20) and the basal cell marker p63, our differentiated urothelial cells did not reproduce an actual bladder urothelial structure. In future studies using our system with transwell culture, the addition of FGF7 to the medium below the insert membrane, which corresponds to the mesenchyme in authentic bladder tissues, might enable us to generate bladder urothelium with a similar expression pattern of uroplakin, CK20 and p63 to authentic bladder urothelium. Furthermore, we should develop stratified urothelial cell sheets suitable for *in vivo* transplantation experiments for clinical application in the future.

In conclusion, we demonstrated for the first time that several factors enhanced the directed differentiation and stratification of bladder urothelium from hiPSCs. Our stratified urothelium model might be useful in the field of regenerative medicine of the bladder as well as studies on the mechanisms underlying the physiology and pathology of the bladder.

## Materials and Methods

### Establishment of a human induced pluripotent stem cell line from peripheral blood mononuclear cells

The human induced pluripotent stem cell (hiPSC) line FF-PB-3AB4 was established from a healthy donor’s peripheral blood mononuclear cells (PBMCs), as described previously^46,47^. In brief, PBMCs were electroporated with the episomal vectors pCXLE-hOCT3/4-shp53 (#27077; Addgene, Cambridge, MA, USA), pCXLE-hSK (#27078; Addgene), pCXLE-hUL (#27080; Addgene), and pCXWB-EBNA1 (Addgene; #37824) using the Nucleofector IIb device (Lonza, Basel, Switzerland) and plated on iMatrix-511-coated cell culture plates (Nippi, Inc., Tokyo, Japan). The iPSCs were induced by changing the medium to StemFit medium (Ajinomoto, Tokyo, Japan). Twenty-nine days after electroporation, colonies were isolated and expanded for validation.

The institutional review board of Kobe University Graduate School of Medicine approved this study (No. 1722), and informed consent was obtained from the donor. FF-PB-3B4 showed typical human embryonic stem cell-like morphology (Fig. S1A), expressed pluripotent stem cell markers OCT3/4 and NANOG (Fig. S1B) and had the ability to differentiate into cells comprising all three germ layers *in vitro* (Fig. S1C). In addition, we performed a “pluritest” and confirmed that the generated clone was pluripotent and similar to validated normal PSCs (Fig. S1D). Karyotype of FF-PB-3AB4 was normal (data not shown).

### Cell culture

FF-PB-3AB4 was maintained with Stem Fit AK02N according to the manufacturer's instructions. Cells were passaged onto precoated with iMatrix-511 (Nippi) at a density of 0.5 mg/cm^2^ every 7 days using 0.5× TripLE select (Life Technologies, Waltham, MA, USA) with 0.5 mM EDTA (Life Technologies) and 10 μM Rho-associated kinase (Rock) inhibitor, Y-27632 (WAKO, Osaka, Japan).

### Definitive endoderm induction

hiPSCs grown with 80% confluency were dissociated into single cells by 0.5× TrypLE select with 0.5 mM EDTA and seeded onto iMatrix-511 coated plates at a density of 1.0 × 10^5^ cells/cm^2^, after which they were cultured for 1 day with Stem Fit AK02N (Ajinomoto) and 10 μM Y-27632. The following day, the medium was changed to RPMI-1640 (NACALAI TESQUE, Kyoto, Japan) containing 100 ng/ml of Activin A (PeproTech, Rocky Hill, NJ, USA), 2% B27 (Life Technologies), 2 mM of L-glutamine (Life Technologies) and 1% Pen strep (Life Technologies). For posterior DE and hindgut induction, CHIR99021 (TOCRIS, Bristol, UK) dissolved in DMSO was added at the following final concentrations: 1, 3, 5 or 7 µM. The medium was changed daily for 3 days. To make the final DMSO concentration consistent, we prepared 1, 3, 5 or 7 mM CHIR dissolved in DMSO and used a 1 in 1000 dilution.

### Differentiation into bladder urothelium

Following DE induction, the medium was changed to RPMI-1640 containing 10 μM all-trans retinoic acid (WAKO) and 100 ng/ml human recombinant FGF10 (R&D Systems, Minneapolis, MN, USA), supplemented with KGM-Gold^TM^ SingleQuots^TM^ (Lonza). The medium was changed every other day for 2 weeks. For terminal differentiation, 1 μM of Troglitazone (Sigma-Aldrich, St. Louis, MO, USA) and 1 μM of PD153035 (Sigma-Aldrich) were added on the last 4 days of this protocol.

### Transwell culture

hiPSCs were seeded onto transwells (0.4-μm pore size; Corning, Corning, NY, USA) pre-coated with iMatrix-511 at a density of 1.0 × 10^5^ cells/cm^2^. The protocol of differentiation was the same as that of normal dish culture.

### Optical coherence tomography

The living cell layers on normal plate and transwell were imaged by Cell^3^iMager Estier (SCREEN Holdings, Kyoto, Japan) without staining. We observed 0.5 mm^2^ with a high-magnification lens and 5 mm^2^ with a low-magnification lens. The X-Y plane resolutions were 1 and 10 μm/pixel, respectively. The Z-axis resolution was 0.92 μm/pixel in both lens. The obtained three-dimensional (3D) imaging data were binarized by the suitable threshold for evaluating the thickness of cell layers using the image processing software program ImageJ (National Institutes of Health, Bethesda, MD, USA). The signal of the well bottom was observed as a high-signal band, and the center of this band was defined as the height reference point of the normal plate. The signal of the transwell membrane was observed as two parallel high-signal bands, indicating the upper and bottom sides of the membrane, and 5 µm (half of the membrane thickness) above the center of these bands was defined as the height reference point of the transwell. Heatmaps were generated using the ImageJ software program, and histograms were plotted using the Python 3.6.5 Seaborn library.

### EdU assay

We used a Click-iT® Plus EdU Alexa Fluor® 555 Imaging Kit (Life Technologies) to evaluate the proliferation of cells. EdU at a final concentration of 10 μM was added, and the cells were incubated at 37 °C for 3 h. The cells were then fixed with only 4% paraformaldehyde. After washing with PBS (−), the cells were permeabilized using PBS (−) containing 0.5% Triton X-100 for 20 min at room temperature and then washed with 3% BSA in PBS (−) after permeabilization and incubated with Click-iT® Plus reaction cocktail, mixed according to the manufacturers’ protocols. After 30-min incubation, protected from light, at room temperature, cells were washed with 3% BSA in PBS (−), and nuclei were stained with Hoechst 33342. The images were taken with a BZ-X700 (Keyence, Osaka, Japan).

### *In vitro* permeability assay

To evaluate the barrier function of urothelium from hiPSCs on transwell plates, we examined the permeability using FITC dextran, a component (Part No. 90328) of the Millipore *In Vitro* Vascular Permeability Assay Kit (Merck Millipore, Billerica, MA, USA). At day 18, the end of differentiation, the medium was carefully removed from the transwell plates without disturbing the cells, and the transwells were transferred to a new plate, where the bottom of the wells was filled with 800 µL of medium. FITC-dextran (300 μL) diluted with differentiation medium (1:40) was then added to each transwell. Samples were incubated for 20 min, protected from light, at room temperature. Permeation of FITC-dextran was stopped by removing the transwells from the wells. The medium containing FITC-dextran in the bottom of the wells was thoroughly mixed, and 100 μL of the medium was transferred into the wells of a black 96-well plate (PerkinElmer, Waltham, MA, USA). Fluorescence intensities were measured using an EnSpire (PerkinElmer), a multi-well microplate reader, with filters appropriate for 490- and 520-nm excitation and emission, respectively.

### Semi-quantitative and real-time quantitative reverse transcription polymerase chain reaction analyses

Total RNA was extracted using TRIZOL regent (Life Technologies) and treated with a TURBO DNA-free kit (Life Technologies), and 500 ng of RNA was reverse transcribed into cDNA using the Prime Script^TM^ II 1st strand cDNA Synthesis Kit (Takara Bio, Shiga, Japan). Semi-quantitative reverse transcription polymerase chain reaction (RT-PCR) was performed with Ex Taq (Takara Bio) on a PCR Thermal Cycler Dice Touch (Takara Bio). The PCR products were run on a 2% (w/v) agarose gel and visualized with ethidium bromide. Real-time quantitative RT-PCR was performed with TB green Premix Ex taq II (Takara Bio) on a LightCycler 480 (Roche, Basel, Switzerland). GAPDH was used as an internal control. Total RNA of human bladder tissue (BioChain Institute Inc., Newark, CA, USA) was used as a positive control. The sequences of the primers used in these studies are described in Table S1.

### Immunohistochemistry

For Uroplakin III, cells were fixed with a mixture of 4% paraformaldehyde and 1% glutaraldehyde for 10 min and then washed with 100 mM glycine. For others, cells were fixed with only 4% paraformaldehyde. After washing with PBS (−), cells were permeabilized and blocked with 1% BSA/PBS (−) containing 0.1% Triton X-100 (NACALAI TESQUE) and 5% normal donkey serum for 1 h at room temperature. The cells were then incubated with the primary antibodies overnight at 4 °C. Primary antibodies and concentrations are listed in Table S2.

Alexa Fluor 488- or Alexa Fluor 594-conjugated secondary antibodies (Life Technologies) were used as secondary antibodies. Nuclei were stained with Hoechst 33342, and the images were taken with a fluorescence microscope (BZ-X700; Keyence). The cells were counted using the hybrid cell count system of the BZ-X700 (Keyence).

### Histological and immunohistochemical analyses of urothelium on transwell

The entire transwell was fixed in 10% buffered formalin, and then the membrane was cut from the well using a scalpel and embedded in paraffin vertically. Immunohistochemistry was performed using the Benchmark XT autostainer (Roche) with an XT ultraView Universal DAB Detection Kit (Ventana Medical Systems, Tucson, AZ, USA), according to the manufacturers’ protocols. The images were obtained with a BZ-X700.

For preparation of cryosections, the fixed membrane was embedded in Tissue-tek O.C.T (Sakura Finetek USA, Torrance, CA, USA) and frozen at −30 °C.

### Embryoid body formation

For embryoid body (EB) formation, undifferentiated iPSCs were dissociated into single cells, resuspended in Primate ES medium (Reprocell, Yokohama, Japan) containing 20 μM Y-27632, and seeded onto low-cell-adhesion 96-well spindle-bottom plates (MS-9096V, PrimeSurface; Sumitomo Bakelite, Tokyo, Japan) at a density of 1.0 × 10^4^ cells per well. After 7 days of culture, the EBs were transferred to gelatin-coated 24-well plates and cultured in the same medium for another 7 days. The differentiated cells were immunostained with the indicated antibodies.

### Pluritest

Pluritest was performed as previously described^48^ using a GeneChip PrimeView Human Gene Expression Array (Thermo Fisher Scientific). The data files were uploaded to www.pluritest.org, scored for pluripotency, and deposited into the National Center for Biotechnology Information (NCBI) Gene Expression Omnibus (GEO) database with accession no. GSM3489481 (201B7), GSM2804015 (409B2) and GSE131371 (3AB4).

All methods were performed in accordance with the relevant guidelines and regulations including Declaration of Helsinki and Ethical Guidelines for Medical and Health Research Involving Human Subjects.

## Supplementary information

Supplemenatry information
