# Supplementary material for: Directed differentiation of human induced pluripotent stem cells into mature stratified bladder urothelium
Source: Sci Rep. 2019 Jul 19;9:10506. doi: 10.1038/s41598-019-46848-8 (PMC6642190; doi:10.1038/s41598-019-46848-8)
Supplement: Supplementary file 1 — Supplemenatry information [file 41598_2019_46848_MOESM1_ESM.docx]

**Supplementary Information**

**Directed differentiation of human induced pluripotent stem cells into mature stratified bladder urothelium**

KOTARO SUZUKI^1,2,3^, MICHIYO KOYANAGI-AOI^1,2,4^, KEIICHIRO UEHARA^1,2,5^,

NOBUYUKI HINATA^3^, MASATO FUJISAWA^3^ and TAKASHI AOI^1,2,4^

1. Division of Advanced Medical Science, Graduate School of Science, Technology and Innovation, Kobe University, Kobe, Japan.

2. Department of iPS cell applications, Graduate School of Medicine, Kobe University, Kobe, Japan.

3. Division of Urology, Graduate School of Medicine, Kobe University, Kobe, Japan.

4. Center for Human Resource development for Regenerative Medicine, Kobe University Hospital, Kobe, Japan.

5. Division of Pathology, Graduate School of Medicine, Kobe University, Kobe, Japan.

**Supplementary Table 1. Primer sets for RT-PCR**

| **Gene** | **Forward** | **Reverse** |
| --- | --- | --- |
| *GAPDH* | ACCACAGTCCATGCCATCAC | TCCACCACCCTGTTGCTGTA |
| *SOX17* | CGCTTTCATGGTGTGGGCTAAGGACG | TAGTTGGGGTGGTCCTGCATGTGCTG |
| *CDX2* | CTGGAGCTGGAGAAGGAGTTTC | ATTTTAACCTGCCTCTCAGAGAGC |
| *HOXA13* | CTGGAACGGCCAAATGTACT | GCTTCTTTCTCCCCCTCCTA |
| *HOXD13* | CACGAGGCCTACATCTCCAT | CCTCTTCGGTAGACGCACAT |
| *Uroplakin IA* | GATCACCAAGCAGATGCTGA | CAGTCCATGGGACCAGATGT |
| *Uroplakin II* | CAGTGCCTCACCTTCCAACA | TGGTAAAATGGGAGGAAAGTCAA |
| *Uroplakin III* | TCCTGAATGCCTACCTGGTC | AAGCCCGTGGACATATTGAC |
| *CK13* | CTGAACAAGGAGGTGTCTACCA | ATAGCGGCACTCCGTCTCT |
| *CK20* | GGTCGCGACTACAGTGCATATTACA | CCTCAGCAGCCAGTTTAGCATTATC |
| *p63* | CTGGAAAACAATGCCCAGA | AGAGAGCATCGAAGGTGGAG |
| *ZO-1* | CGAGTTGCAATGGTTAACGGA | TCAGGATCAGGACGACTTACTGG |
| *E-Cadherin* | CCCGGGACAACGTTTATTA | GCTGGCTCAAGTCAAAGTCC |

RT-PCR, reverse transcription polymerase chain reaction

**Supplementary Table 2. Primary antibodies for immunostaining**

| **Antibody** | **species** | **Dilution** | **Source** |
| --- | --- | --- | --- |
| SOX17 | goat | 1:200 | AF1924, R&D Systems |
| CDX2 | mouse | 1:100 | 39-7800, Invitrogen |
| HOXA13 | rabbit | 1:200 | ab106503, Abcam |
| HOXD13 | rabbit | 1:200 | ab19866, Abcam |
| CK13 | rabbit | 1:200 | ab92551, Abcam |
| CK20 | mouse | 1:200 | ab854, Abcam |
| Uroplakin II | mouse | ready to use | 418121, Nichirei |
| Uroplakin III | rabbit | 1:100 | ab93721, Abcam |
| OCT3/4 | mouse | 1:100 | 611202, BD Transduction Laboratories |
| Nanog | goat | 1:50 | AF1997, R&D Systems |
| α-SMA | mouse | 1:500 | M0851, DAKO |
| βIII-tubulin | mouse | 1:500 | MAB1637, Millipore |
| p63 | goat | 1:200 | BAF1916, R&D Systems |
| ZO-1 | mouse | 1:100 | 33-9100, Thermo Scientific |
| E-Cadherin | goat | 1:200 | AF548, R&D Systems |
| Caspase-3 | rabbit | 1:400 | 9664, Cell Signaling Technologies |

**
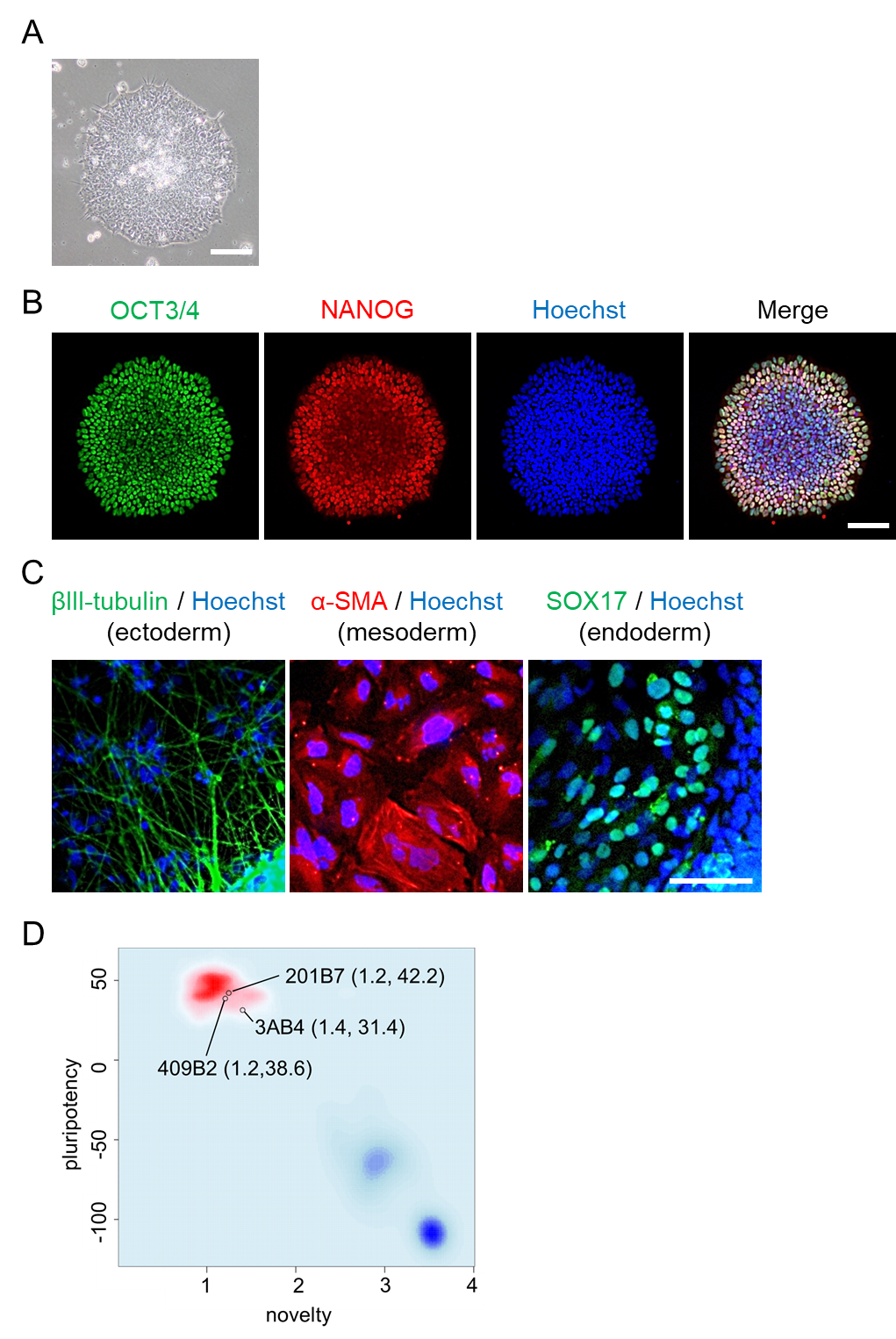
Supplementary Figure 1. The characterization of the hiPSC line PB-3AB4 used in this study.** (A) A phase contrast image of hiPSC PB-3AB4 colony before differentiation. Scale bar, 100 μm. (B) An immunofluorescence analysis of the hES cell markers OCT3/4 (green) and NANOG (red) in PB-3AB4. Nuclei were stained with Hoechst 33342 (blue). Scale bar, 100 μm. (C) *In vitro* differentiation via embryoid body formation of PB-3AB4. The images show immunofluorescence staining for β III-tubulin (ectodermal marker, left panel), α-smooth muscle actin (mesodermal marker, middle panel), and SOX17 (endodermal marker, right panel) in iPSC-derived differentiated cells. The nuclei were stained with Hoechst 33342 (blue). Scale bar, 50 μm. (D) The PluriTest results of 3AB4 and conventional iPSC lines (201B7 and 409B2). 3AB4 was pluripotent and similar to validated normal hPSCs; this was indicated by a Pluripotency Score of >20 (y-axis) and a Novelty Score of <1.67 (x-axis).

**
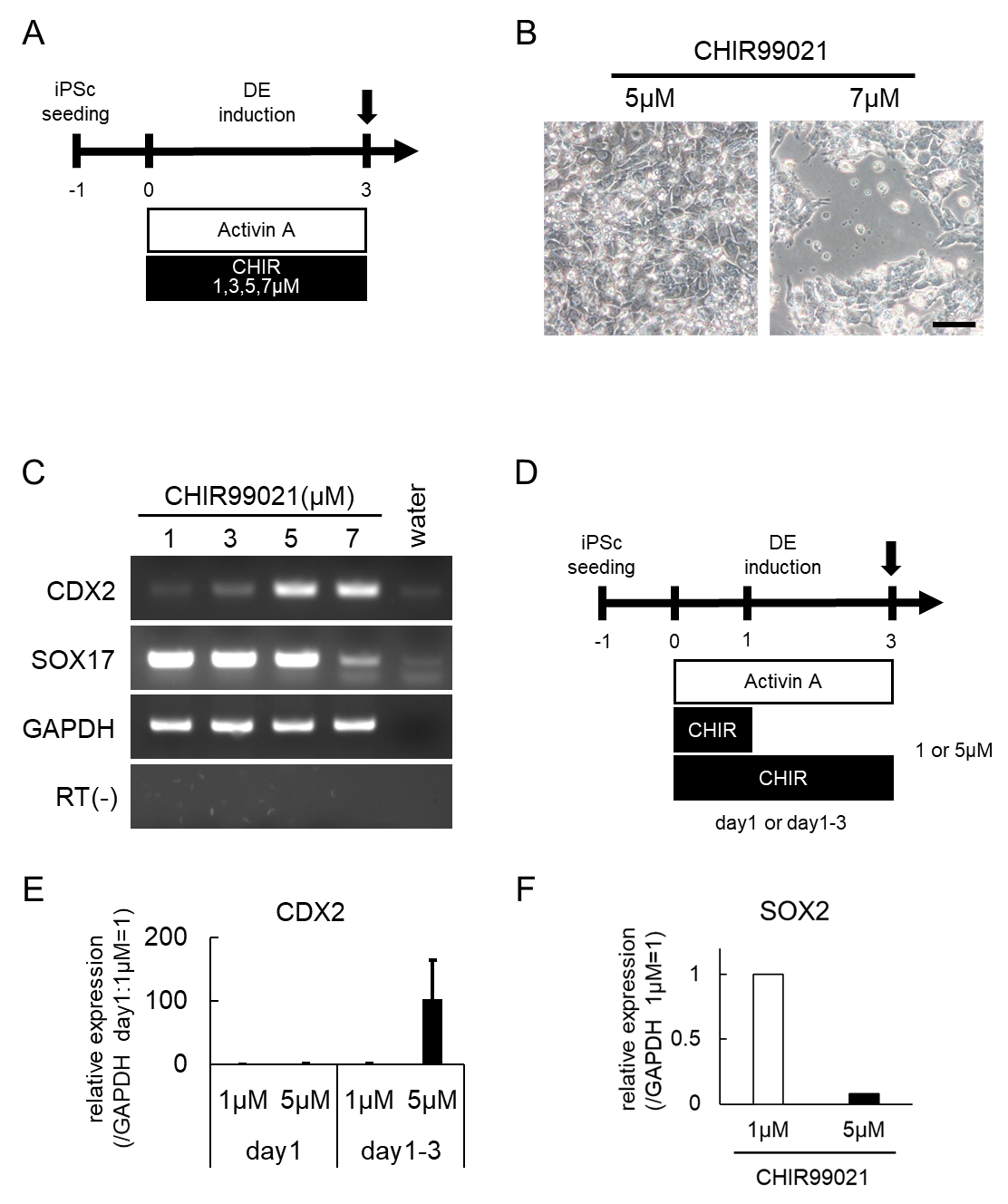
Supplementary Figure 2. Effects of CHIR99021 treatment on posterior DE induction.** (A) An overview of the culture protocol for posterior DE induction with the indicated dose of CHIR. (B) Phase contrast images of differentiated cells after treatment with 5 µM (left panel) or 7 µM (right panel) CHIR for 3 days. Scale bar, 50 µm. (C) An expression analysis of SOX17 and CDX2 by semi-quantitative RT-PCR. GAPDH was used as a loading control. Full-length gels are presented in Supplementary Figure 8. (D) An overview of the culture protocol for posterior DE induction with the indicated duration of CHIR treatment. (E) An expression analysis of CDX2 in differentiated cells treated with the indicated dose of CHIR for the indicated duration by qPCR (n=3 independent experiments; mean ± SE). (F) An expression analysis of SOX2 in differentiated cells treated with the indicated dose of CHIR by qPCR. Abbreviations: iPSc, induced pluripotent stem cell; DE, definitive endoderm; CHIR, CHIR99021.


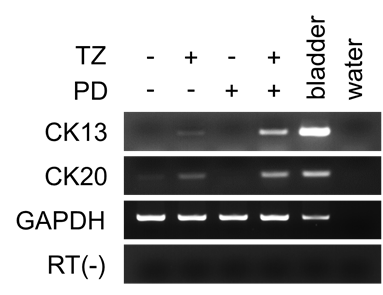


**Supplementary Figure 3. Effects of a PPAR-γ agonist and EGFR inhibitor on terminal differentiation.** An expression analysis of CK13 and CK20 in the cells treated with either Troglitazone or PD153035, or both by semi-quantitative RT-PCR. GAPDH was used as a loading control. Full-length gels are presented in Supplementary Figure 9. Abbreviations: TZ, Troglitazone; PD, PD153035.

**
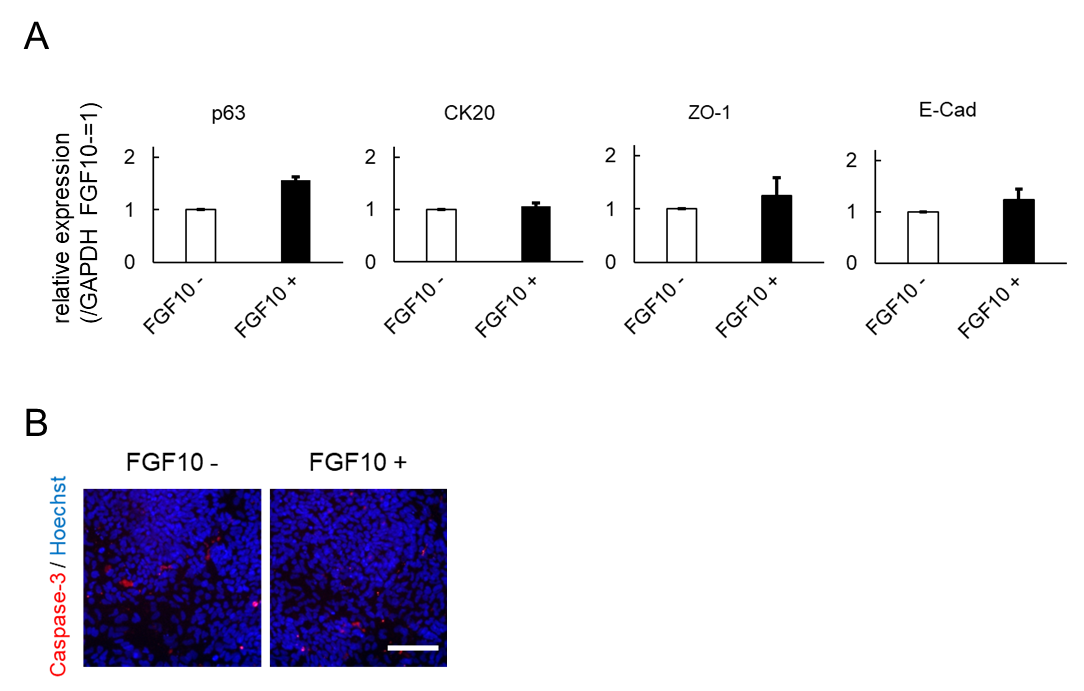
**

**Supplementary Figure 4. Effects of FGF10 on urothelial differentiation.** (A) An expression analysis of p63, CK20, ZO-1 and E-Cadherin in differentiated cells with or without FGF10 treatment by qPCR (n=3 independent experiments; mean ± SE). (B) An immunofluorescence analysis of Caspase-3 (red). Nuclei were stained with Hoechst 33342 (blue). Scale bar, 100 µm.


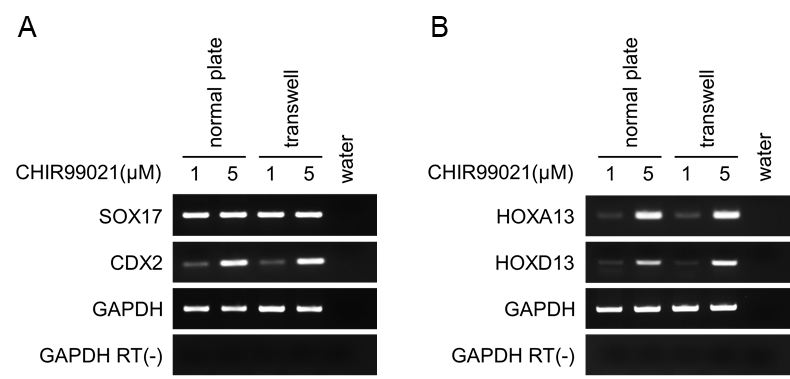


**Supplementary Figure 5. Effects of transwell culture on urothelial differentiation.** Semi-quantitative RT-PCR for the expression analysis of (A) CDX2 and SOX17 at day 3 and (B) HOXA13 and HOXD13 at day 7 in normal plates and transwell plates, in which cells were treated with 1 or 5 µM of CHIR99021. GAPDH was used as a loading control. Full-length gels are presented in Supplementary Figure 10.


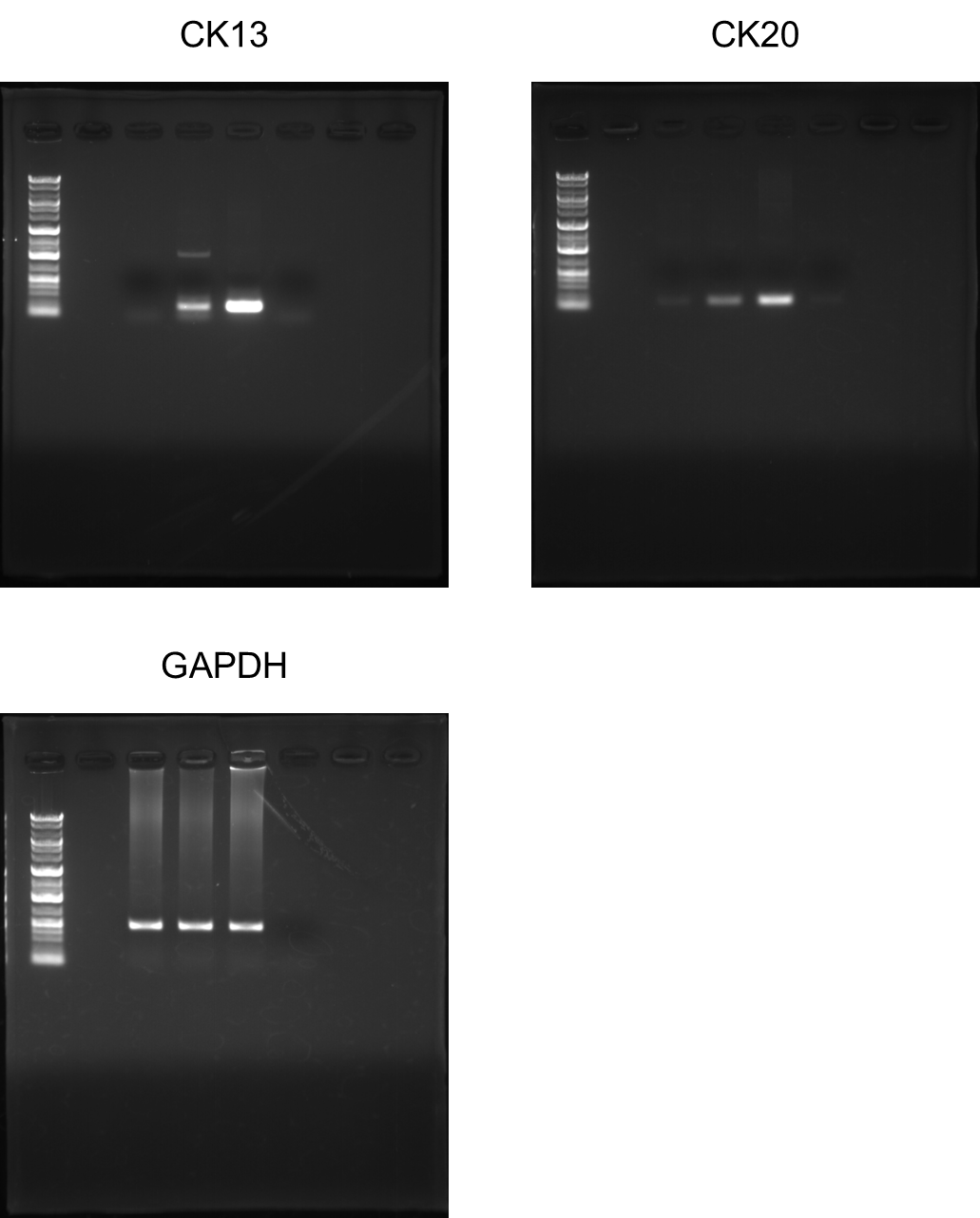


**Supplementary Figure 6.** An expression analysis of the transitional differentiation markers CK13 and CK20 at day 18 by semi-quantitative RT-PCR. Figure 3C was cropped from the above gels.

**
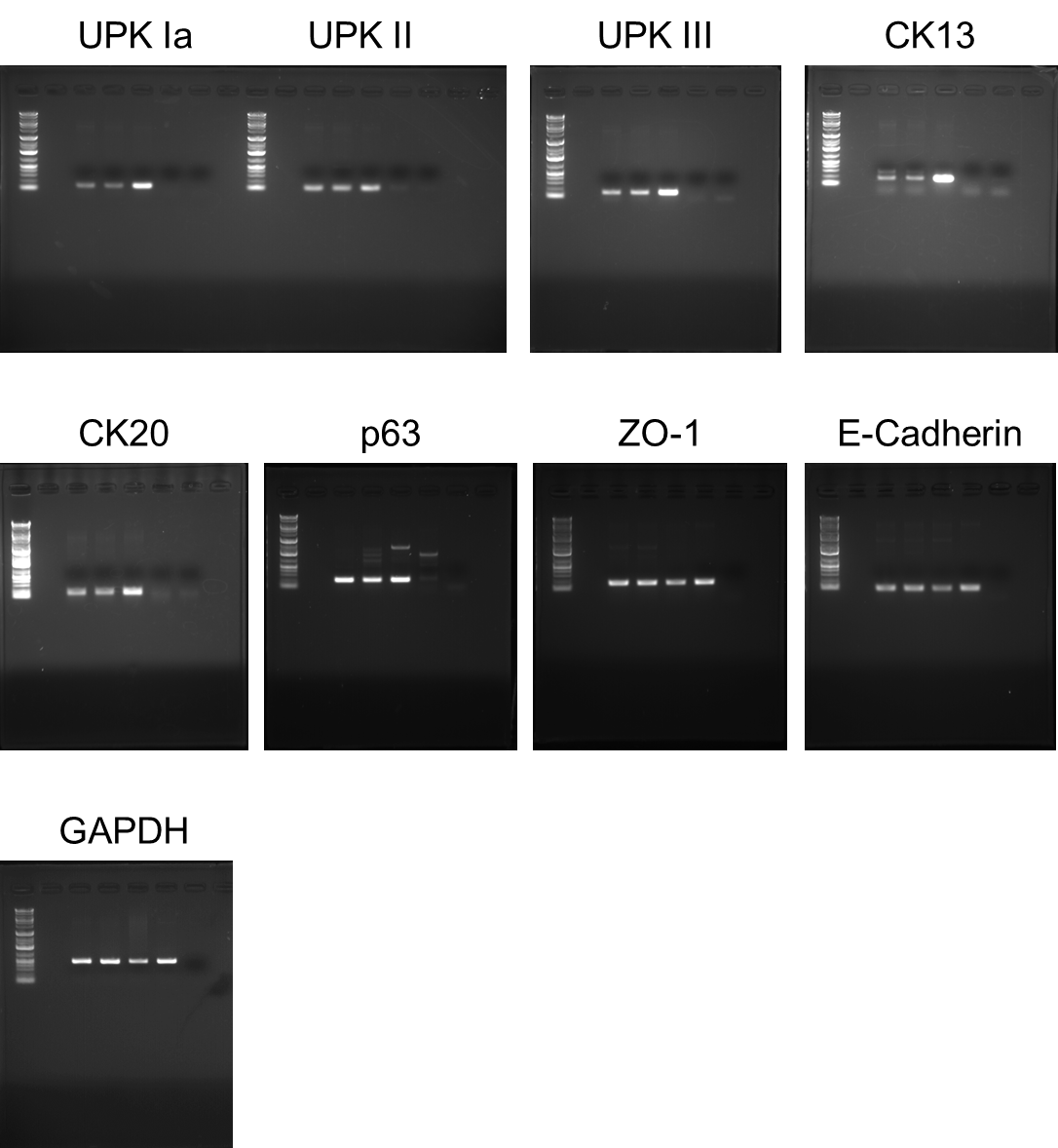
**

**Supplementary Figure 7.** A semi-quantitative RT-PCR analysis of urothelial markers (UPK Ia, UPK II, UPK III), transitional differentiation markers (CK13, CK20) and other differentiation markers (p63, ZO-1, E-Cadherin) in differentiated cells cultured on normal plates and transwells. Figure 6C was cropped from the above gels.

**
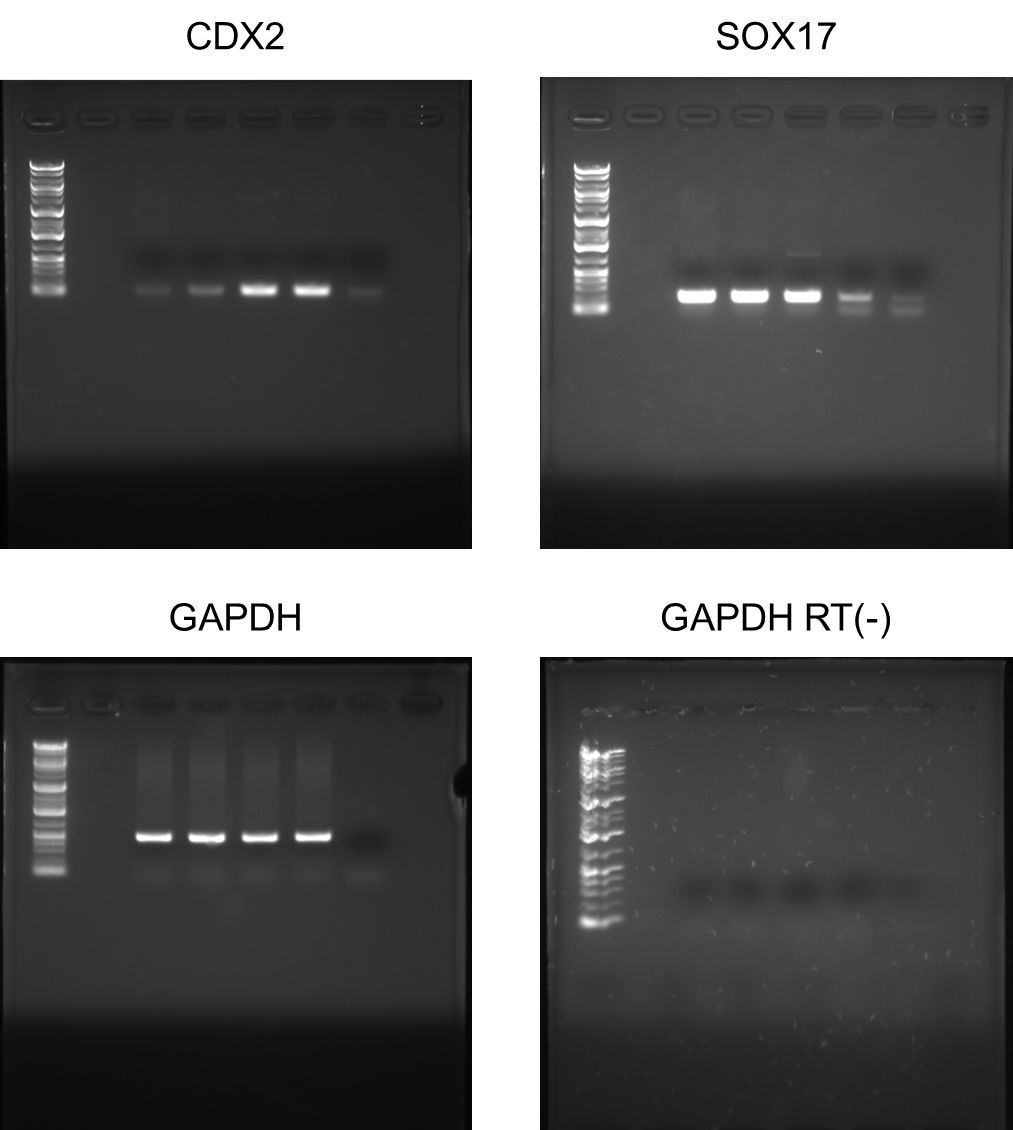
**

**Supplementary Figure 8.** An expression analysis of SOX17 and CDX2 by semi-quantitative RT-PCR. Figure S2C was cropped from the above gels.


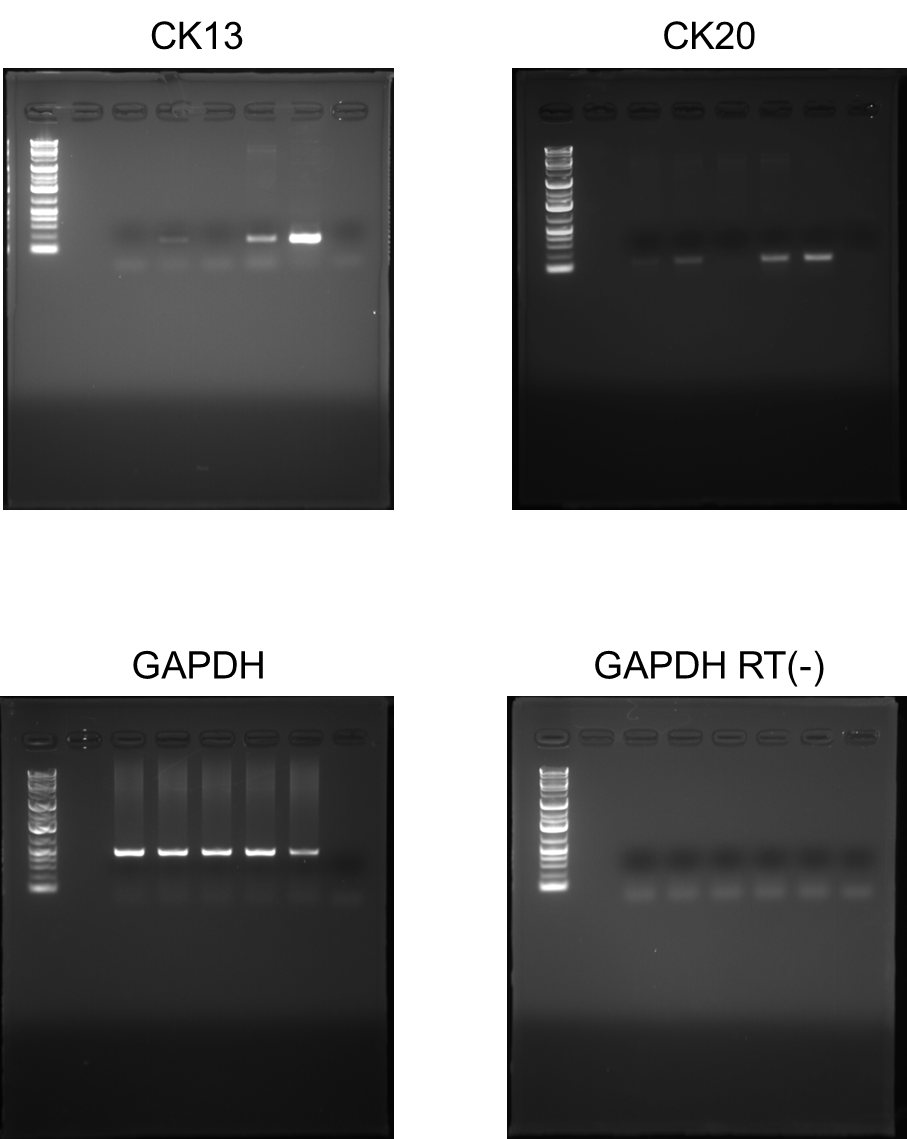


**Supplementary Figure 9.** An expression analysis of CK13 and CK20 by semi-quantitative RT-PCR. Figure S3 was cropped from the above gels.

**
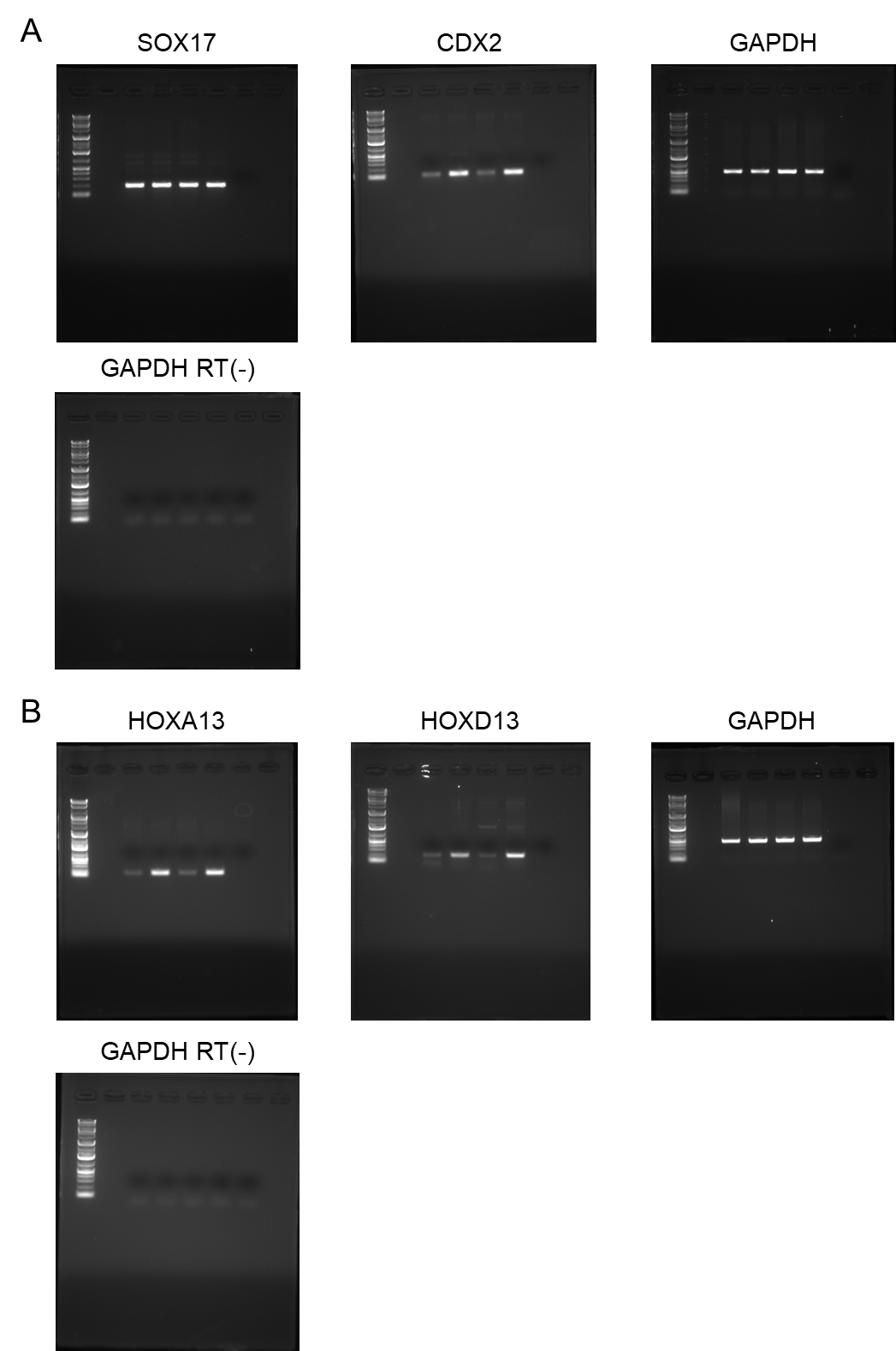
**

**Supplementary Figure 10.** (A) An expression analysis of SOX17 and CDX2 by semi-quantitative RT-PCR. (B) An expression analysis of the hindgut markers HOXA13 and HOXD13 by semi-quantitative RT-PCR. Figure S5A and B were cropped from the above gels.
